# UK Biobank: opportunities for cardiovascular research

**DOI:** 10.1093/eurheartj/ehx254

**Published:** 2017-05-20

**Authors:** Thomas J Littlejohns, Cathie Sudlow, Naomi E Allen, Rory Collins

**Affiliations:** 1 Clinical Trial Service Unit and Epidemiological Studies Unit, Nuffield Department of Population Health, University of Oxford, Richard Doll Building, Old Road Campus, Oxford, UK; 2 Centre for Clinical Brain Sciences, University of Edinburgh, Chancellor's Building, 49 Little France Crescent, Edinburgh, UK

## Introduction

Cardiovascular diseases are a major cause of morbidity and mortality, accounting for 45% of all deaths in European countries in 2016^1^ and almost a third of deaths worldwide in 2013.^2^ A similar pattern is observed in the UK where cardiovascular diseases were responsible for 27% of deaths in 2014, with coronary heart disease resulting in the largest number of deaths attributable to a single cause (*n* = ∼69 000) whilst stroke is the third biggest cause (*n* = ∼39 000).^3^ Although age-standardized cardiovascular disease mortality rates are decreasing worldwide, the total deaths and burden as measured through disability-adjusted life years of cardiovascular diseases are increasing.^4^^,^^5^ Furthermore, in the UK, cardiovascular risk factors such as high blood pressure and high cholesterol are among the leading causes of disease burden.^6^

Epidemiological studies have historically played an essential role in identifying the causes and consequences of cardiovascular disease and have resulted in improvements in prevention and treatment. The seminal US-based Framingham Heart Study which recruited 5200 participants between 1948 and 1952, was integral in identifying a range of important risk factors for cardiovascular disease, such as high blood pressure, a high cholesterol level, cigarette smoking, obesity and physical inactivity, and consequently shifted the focus from management to preventative strategies for cardiovascular disease.^7^ This, together with findings from other epidemiological studies, such as the Seven Countries Study and the MONICA project,^8^ have been influential in leading to treatments for the primary and secondary prevention of cardiovascular events, most notably statins (that act to lower cholesterol levels), and anithypertensives.^9^^,^^10^

**Figure 1:**
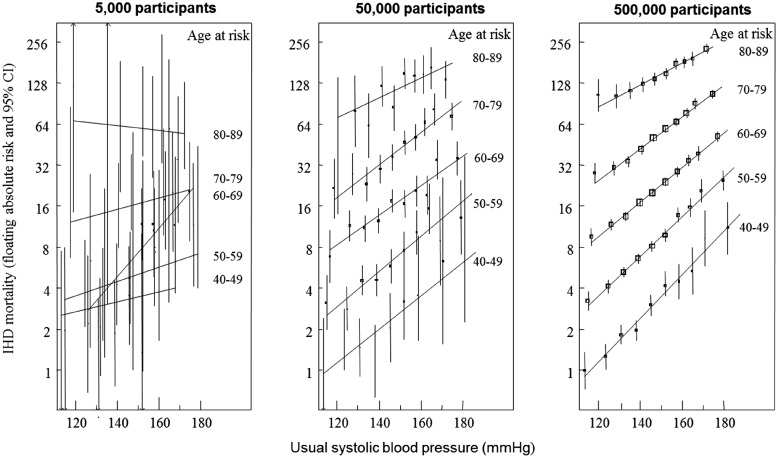
Absolute risk of ischaemic heart disease mortality by usual systolic blood pressure and age at risk in 5000, 50 000, and 500 000 participants. Unpublished figure containing data from the Prospective Studies Collaboration, obtained through personal communication. CI, confidence interval; IHD, ischaemic heart disease.

Epidemiological studies such as the Framingham Heart Study with moderate sample sizes are useful in detecting risk factors with large effects on common outcomes; however, they lack statistical power to reliably identify risk factors which have small to moderate effects or to assess associations with disease across subgroups of the population. The need for large sample sizes has led to collaborative efforts, such as the Prospective Studies Collaboration (an individual participant meta-analysis of data from 61 studies and more than a million participants^11^) that has demonstrated conclusively that a continuous increase in blood pressure corresponds with an increased risk of vascular death across all age groups (see *Figure **1* that illustrates the importance of a large sample size (about 500 000 participants) for detecting this association).^12^ Sample size is also of particular importance in the current era of genome-wide association studies, where many investigations are aiming to detect either small effects from common variants or large effects from rare variants.^13^

The causes of cardiovascular disease involve a complex interplay between predisposing genetic factors and lifestyle, environmental, and health-related exposures. Furthermore, cardiovascular risk factors are likely involved in the development of non-cardiovascular diseases, such as Alzheimer’s disease.^14^ Large prospective studies that collect an extensive range of exposures before the subsequent development of disease are essential in order to gain novel insights into the causes and consequences of cardiovascular (and non-cardiovascular) diseases. Although there are many cohort studies with large sample sizes and biological samples, they generally consist of less comprehensive data collection (see *Table **1* for overview of major studies). In order to address this, UK Biobank was established as a prospective cohort study that combines a large sample size with a very wide range of data on exposures and outcomes in order to improve the prevention, diagnosis and treatment of diseases of middle and old age, including cardiovascular diseases such as heart disease and stroke.^15–17^

**Table 1:** Characteristics of major population-based prospective cohort studies with biological samples for 100 000 or more participants

Study	Initial scientific focus	Study details	Physical measures	Repeat assessment	Samples^a^	Genotyping^a^	Imaging^a^	Follow-up health-record linkage^a^
China Kadoorie Biobank	To explore the interplay between lifestyle, environmental and genetic factors and the risk of chronic diseases	510 000 men and women aged 30–79 recruited from 10 regions in China (5 urban, 5 rural) between 2004–08	Yes	In subsets of 25 000 every few years	Blood sample; Urine sample for 25 000 participants at second repeat assessment	Candidate array (384 SNPs) and GWAS array (800 000 SNPs) on ∼100 000 participants	cIMT, bone mineral density and ECG on 25 000 participants at second repeat assessment	Mortality registry; stroke, IHD, cancer, and diabetes registries; hospital admissions
European Prospective Investigation into Cancer and Nutrition (EPIC)	To investigate dietary, lifestyle and environmental factors in relation to cancer and chronic disease incidence	520 000 men and women mostly aged 35–70 recruited in 23 centres in 10 European countries between 1992–99	Types of measures varied by centres	Recontact every 3–5 years for lifestyle factors	Blood samples for 385 000 participants	Available for nested case-control subsets	No	Cancer registry in seven countries; combination of health insurance records cancer and pathology registries, and by active follow-up through participants and next-of- kin in three countries; Mortality registries or active follow-up and death-record collection; hospital admission data available in some countries
Lifelines	To investigate interactions between environmental, phenotypic and genetic factors in the development of chronic disease and healthy ageing	167 000 adults and children (aged 6+) recruited from 3 provinces in the Netherlands between 2006–13	Yes	Plans to invite all participants every 5 years	Blood and urine	GWAS array (270 000 SNPs) on 15 000 participants	No	Planned linkages to GP and hospital admission records
Million Veteran Program	A national representative and longitudinal study for genomic (and non-genomic) research	Recruitment began in 2011 and is ongoing. (As of August 2015, 397 000 men and women mostly aged ≥50 years recruited across the USA)	No	No	Blood samples	GWAS array (675 000 SNPs) on 199 000 participants (ongoing)	No	Electronic health records
Kaiser Permanente Research Bank	Developed to facilitate research on genetic and environmental factors on common diseases and healthy aging	Recruitment began in 2007 with the aim of recruiting 500 000 men and women aged ≥18 from the Northern California region in the USA	No	No	Blood and saliva samples	GWAS array (675 000 SNPs)	No	Hospitalizations Clinic visits Laboratory testing results Pharmacy Dispensing Records

cIMT, carotid intima-media thickness test; ECG, electrocardiogram; GWAS, genome-wide association study; IHD, ischaemic heart disease; NHS, National Health Service; SNP, single nucleotide polymorphism.

a Cohort-wide coverage unless otherwise stated.

## UK Biobank

Between 2006 and 2010, half a million participants aged 40–69 years who lived within ∼25 miles of one of the 22 assessment centres located throughout England, Wales and Scotland were recruited into UK Biobank. At the assessment centres, participants provided electronic signed consent, answered touchscreen and verbal interview questions on sociodemographic, lifestyle, environmental, and health-related factors, completed a range of physical measures and provided blood, urine, and saliva samples (see *Table **2* for further detail on measures collected at baseline). Once recruitment was fully underway, further enhancements were introduced to the assessment visit, with large subsets of the cohort undergoing a range of eye measures, heel bone ultrasound, an electrocardiograph test, pulse wave velocity, and a hearing test. A large amount of the data collected at baseline has direct relevance to cardiovascular disease and health, including, but not limited to, self-reported information on medications and health conditions, family history of cardiovascular disease, measures of arterial stiffness, blood pressure, cardiorespiratory fitness, body size, and body fat.

**Table 2:** Overview of data collected at baseline assessment in 2006–10

Topics/measures	Details
Touchscreen questionnaire and computer assisted verbal interview	
Sociodemographics	Ethnicity, education, employment, household information, Townsend deprivation index (social class)
Lifestyle and environment	Smoking, alcohol consumption, physical activity, diet, sleep, electronic device use, sun exposure, and sexual factors
Early life factors	Birthplace, birth weight, breastfed, childhood body size and height, maternal smoking, handedness, adopted, and part of multiple birth
Family history	Illnesses of father/mother/siblings, age of parents, age parents died, and number of siblings
Psychosocial factors	Social support, bipolar/major depression, anxiety, nerves, psychological traits, and mood
Health and medical history	Medical conditions, medications, operations, cancer screening, pain, oral health, eyesight, hearing, and general health
Sex-specific factors	Male specific—first facial hair, age voice broke, hair/balding pattern, children fathered; female specific—hormone-replacement therapy, contraception, pregnancy, menstruation, menopause, and cervical test
Cognitive function	Prospective memory^a^, pairs matching, fluid intelligence^a^, reaction time, and numeric memory^b^
Hearing test^a^	Speech-in-noise
Physical measures	
Blood pressure and pulse rate	Two measures taken 1 min apart using a digital blood pressure monitor
Arterial stiffness^a^	Pulse wave velocity using infra-red sensor at the finger
Grip strength	Right and left hand isometric grip strength
Anthropometrics	Standing/sitting height, waist/hip circumference, weight body mass index, and whole body bio-impedance measures
Spirometry	Two to three blows within a 6 min period
Bone mineral density	Ultrasound measurement of the heel
Eye measures^c^	Eye surgery complications, visual acuity, autorefraction, intraocular pressure, and retinal coherence tomography
Fitness test^c^	Heart rate monitoring using a four-lead electrocardiograph during cycle ergometry on a stationary bike
Sample collection	
Blood	45 mL divided into 6 tubes, includes whole blood, serum, plasma, red cells, buffy coat
Urine	9 mL in 1 tube
Saliva^c^	2.5 mL in 1 tube

a Performed in last 200 000 participants.

b Performed in 50 000 participants (as part of the pilot study).

c Performed in last 100–150 000 participants.

The cohort is not representative of the general population (e.g. participants are more likely to live in less socio-economically deprived areas and have lower death rates than the general population), so is unsuitable for estimating disease prevalence and incidence rates. However, it is well-designed to reliably detect generalizable associations between most baseline characteristics and health outcomes due to the sufficiently large numbers of participants across the full distribution of exposures. For example, whilst the number of current smokers is low in UK Biobank compared with the general population, there are sufficiently large numbers of smokers to detect the association with various diseases.

### Enhancements to data collection

The UK Biobank resource is continuously being enhanced through additional phenotyping (see *Table **3* for further detail on ongoing data collection). The samples from all 500 000 participants are currently being assayed for a range of selected biomarkers, many of which have been implicated in the development of cardiovascular disease (e.g. cholesterol, direct low-density lipoprotein, high-density lipoprotein, lipoprotein (a), triglyceride, apolipoprotein A, apolipoprotein B, and C-reactive protein).

**Table 3:** Overview of ongoing data collection and enhanced phenotyping

Data	Details	Date collected	Date available for research use
Genotyping	Blood collected at baseline for the full cohort has been genotyped by two arrays that share 95% common content (the UK BiLEVE array for 50 000 participants and the UK Biobank Axiom array for 450 000 participants). The array covers ∼800 000 SNPs and indel markers covering markers of specific interest, rare coding variants and genome-wide coverage. Seventy-three million SNPs, short indels, and large structural variants have been imputed using the UK10K haplotype reference panel merged with the 1000 Genomes Phase 3 reference panel. For more detail, see: http://biobank.ctsu.ox.ac.uk/crystal/exinfo.cgi?src=GeneticData.	Samples collected in 2006–10	150 000 in Q4 2015; 350 000 in Q2 2017
Biochemical measures	Thirty-four biomarkers are being assayed using the plasma, serum, red blood cells, and urine samples. Biomarkers were selected because they are established risk factors for disease (e.g. sex hormones for cancer), diagnostic measures (e.g. HbA1C for diabetes) or they are used to characterize phenotypes (e.g. cystatin C and creatinine for renal function). For more detail, see: http://biobank.ctsu.ox.ac.uk/showcase/catalogs.cgi..	Samples collected in 2006–10	Urinary biomarkers in Q4 2016; Red blood cells and serum biomarkers in Q3–Q4 2017
Repeat of baseline assessment	Twenty-thousand participants repeated all baseline assessment measures at one assessment centre, Stockport, UK. These will be repeated every few years.	2012–13	Q3 2013
Web-based questionnaires	Participants with email addresses (∼330 000) are sent web-based questionnaires once or twice a year to collect more detailed information on exposures or health outcomes that are difficult to capture through linkage to electronic health records.		
24-h dietary recall	Includes information on consumption of over 200 food and drink items over the last 24 h and was used to generate estimated daily nutrient intakes. The questionnaire was sent on four occasions over a 16-month period to capture variation in diet. 176 012 participants completed the questionnaire at least once (53% response rate) and 27 535 completed it four times (16%).	2011–12	Q2 2013
Cognitive function	Includes a series of cognitive tests, of which four were repeated from the baseline assessment (fluid intelligence, reaction time, numeric memory, pairs test) in addition to two further tests (trail making, symbol digit substitution). 120 000 participants completed this questionnaire (36% response rate).	2014	2015
Occupational history	Included information on lifetime employment history, occupational exposures and related medical information. 117 500 participants completed this questionnaire (35% response rate).	2015–16	2015
Mental health	Included information on lifetime mental health events (including depression, bipolar affective disorder, and generalized anxiety disorder), alcohol and cannabis use, unusual and psychotic experiences, traumatic events, self-harm behaviours and subjective wellbeing. 137 400 participants completed this questionnaire (45% response rate).	2016	Q2 2017
Accelerometry	100 000 participants wore an Axivity AX3 tri-axial wrist accelerometer for a 7-day period. Derived summary data on duration and intensity of activity available.	2013–15	2015
Multi-modal imaging	MRI of brain, heart and body, carotid ultrasound and whole body DXA scan of bones and joints for 100 000 participants.	Pilot phase: 2014–15; Main phase: 2015–20	2015—ongoing

DXA, Dual-energy X-ray absorptiometry; MRI, magnetic resonance imaging; SNP, single nucleotide polymorphism.

**Figure 2:**
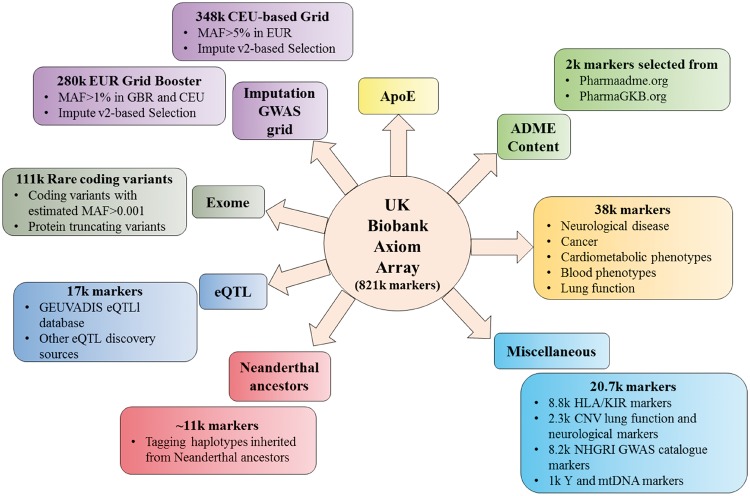
Overview of the genetic markers measured on the UK Biobank Axiom Array. ADME, absorption, distribution, metabolism, and excretion; ApoE, apolipoprotein E; CNV, copy number variants; eQTL, expression quantitative trait loci; GWAS, genome-wide association study; HLA, human leucocyte antigen; k, thousand; KIR, killer cell immunoglobulin-like receptors; MAF, minor allele frequency; mtDNA, mitochondrial DNA; NHGRI, National Human Genome Research Institute.

Genotyping of 820 000 single nucleotide polymorphisms and insertion-deletion markers has been performed using a bespoke genome-wide array, designed collaboratively by a group of leading academics and Affymetrix, with centralized quality control and imputation to >70 million variants. The genotyping array includes thousands of markers that are involved in cardiometabolic processes and blood pressure regulation, rare variants associated with cardiac disease and those involved in the absorption, distribution, metabolism, and excretion of drugs (*Figure **2*).^18^ This data will enable researchers to explore the genetic determinants of cardiovascular disease, conduct Mendelian randomization experiments to identify potentially causal effects and investigate gene-environment interactions.

Subsets of the cohort are invited to have a repeat assessment every few years (the first of which was performed during 2012–13 on 20 000 participants) to allow for correction for regression dilution bias caused by measurement error or intra-individual changes in exposures and biomarkers.^19^

**Figure 3:**
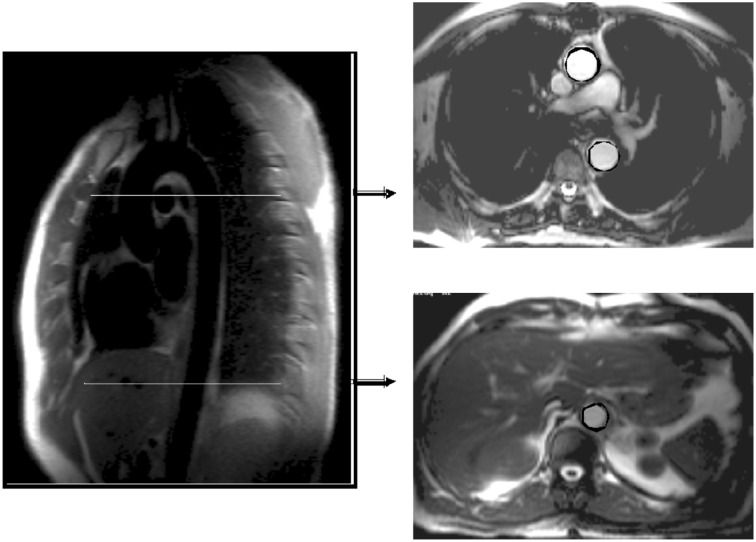
Plane selection for measures of distensibility and pulse wave velocity in ascending, proximal, descending, and distal descending aorta obtained from cardiac magnetic resonance imaging (measured at the Oxford Centre for Clinical Magnetic Resonance Research).

UK Biobank is undertaking multimodal imaging in 100 000 participants.^20^ Imaging measures relevant for cardiovascular research include cardiac magnetic resonance imaging (MRI), which measures the left and right ventricles and atrium, aorta and aortic valve (see *Figure **3* e.g. of cardiac MRI images performed in UK Biobank),^21^ an ultrasound of the carotid arteries^22^ and a resting 12-lead electrocardiogram (ECG).^23^ The other imaging modalities include an MRI of the brain and body and a whole body dual-energy X-ray absorptiometry (DXA) of the bones and joints. These modalities also capture information of relevance to cardiovascular health, e.g. white matter lesions on T2-weighted brain MRI scans as well as fat distribution from liver and pancreatic MRI scans.

Analytical pipelines are being set up so that derived phenotypes, such as detailed measures of brain structure and function, body fat distribution and cardiac function can be made available for researchers. The imaging assessment began in 2014 with a pilot study of 6000 participants and is now being expanded to three assessment centres over the next few years. Imaging data will be released in tranches every 6–12 months as it becomes available so researchers can continuously refresh their analyses throughout the course of their project without having to wait until the end of the imaging study (2020 or later).

Data are also being collected from web-based questionnaires that focus on the collection of more detailed information on exposure (such as dietary habits and occupational history) and of outcomes that are difficult to ascertain through electronic health records (such as cognitive function, mental health, irritable bowel syndrome, pain, and quality of life).

### Capturing health outcomes through data linkage

A major advantage of UK Biobank is that all participants at recruitment were registered with a general practitioner in the National Health Service (NHS) and consented to linkage to their health-related records. The NHS provides nationwide healthcare in the UK and keeps detailed records of health-related information. As a result, UK Biobank can follow-up all participants’ health outcomes through linkage to a range of national datasets. Currently, data from national death and cancer registries and hospital inpatient records are available whilst efforts are underway to integrate data from primary care, screening programmes, and disease-specific registries (See *Table **4* for more detail on health-records).

**Table 4:** Overview of linkage to health-related records

Data	Details	Date of coverage
Death	ICD-10 coded national death registry data obtained from the Health and Social Care Information Centre (now NHS Digital) for England and Wales and the Information Services Department (ISD) for Scotland. Contains information on source of death report, date, age and cause(s) of death	2006—ongoing
Cancer	ICD-9 and -10 coded national cancer registry data obtained from HSCIC for England and Wales and the ISD for Scotland. Contains information on source of cancer report, date and age at diagnosis, site, histology, and behaviour of the cancer. Detailed data on stage and grade of tumours will be made available in Q3–Q4 2017	England and Wales: 1995—ongoing Scotland: 1957—ongoing
Inpatient hospital admissions	ICD-9 and -10 coded hospital inpatient episodes obtained from the Hospital Episode Statistics provider for England, the Patient Episode Data for Wales and the Scottish Morbidity Records for Scotland. Contains information on admission and discharge, operations, diagnoses, maternity care, and psychiatric care. Main and secondary diagnoses/operations as well as date of diagnosis/operation are included.	England: 1996—ongoing Wales: 1998—ongoing Scotland: 1997—ongoing
Primary Care	Coded data from primary care records, including diagnoses, prescriptions, referrals etc. will be made available 2017–18	Lifetime—ongoing
Other	Efforts are ongoing to link to additional external datasets including hospital outpatient admissions, screening programmes, and disease-specific registries and others.	Not applicable

The authors do hereby declare that all illustrations and figures in the manuscript are entirely original and do not require reprint permission.

Linking to a wide range of health record datasets will provide a rich amount of data. However, it also introduces the daunting challenge of harmonizing information across multiple sources to produce reliable and valid outcomes. In order to ensure effective use of the resource, UK Biobank aims to develop scalable approaches not only for the ascertainment of the many thousands of many different health-related outcomes that will occur during prolonged follow-up (as well as those that occurred prior to recruitment) but also for their sub-classification and detailed characterization. This will be achieved through a staged approach of initially ascertaining cases using linked data, then increasing the accuracy of diagnoses by cross-referencing multiple sources of information, starting with the ascertainment of health outcomes using lower-cost linked health-related data sources,^24^^,^^25^ and proceeding to more intensive methods (e.g. retrieval of imaging or laboratory data) for validation and sub-classification.

Expert-led health outcome subgroups are guiding the development and testing of approaches for a wide range of conditions (including cardiac diseases, stroke, diabetes, musculoskeletal, neurodegenerative, and mental health disorders, renal and eye diseases). These adjudicated outcomes will provide an invaluable resource for researchers interested in investigating cardiovascular disease, or non-cardiovascular diseases in the context of cardiovascular research, but who do not have the time or expertize to derive these outcomes themselves. Alternatively, researchers who are interested in developing their own algorithms to enhance the classification of diseases or extract useful information from the medical records have access to an increasing diversity of linked data.

## UK Biobank and implications for cardiovascular health and disease research

Observational studies tend to focus on either collecting a diverse range of measures from a small number of participants or less detailed information on a large number of participants. In contrast, UK Biobank combines a large sample size with extensive phenotypic and genotypic data as well as ongoing follow-up of participant’s health through linkage to electronic medical records. The obvious advantage of conducting research on a single large well-phenotyped population is that the assays and measurements have been performed in a standardized way as opposed to integrating data from multiple studies consisting of widely varying participant characteristics and different methodologies.

The unprecedented depth and breadth of data available in UK Biobank offers unparalleled opportunity to address a wide range of research questions related to cardiovascular health outcomes. Classification and sub-classification of diseases can be enhanced through combining the diverse phenotypic and genotypic data with medical records. The large sample size enables researchers to perform risk stratification on well-defined phenotypes to focus on high- and low-risk populations for cardiovascular disease, e.g. those with the lowest and highest levels of circulating lipid levels. Additionally, mechanistic pathways between risk factors and outcomes can be explored using the genetic, biomarker and imaging data. UK Biobank is already the largest-ever multimodal imaging study; previous studies that have incorporated cardiovascular imaging have usually included a few thousand participants, which has limited the potential research opportunities available.^20^ UK Biobank will provide sufficient statistical power to investigate imaging derived phenotypes in association with a range of incident health outcomes, as well as the interplay between the heart, body and brain in determining disease risk.

UK Biobank has also included a range of physical measures to complement self-reported information which is prone to various biases, e.g. the collection of objective physical activity on 100 000 participants using accelerometers, allowing the quantification of the type and amount of physical activity in association with cardiovascular health, as well as the relationship with other factors such as sedentary behaviour, obesity, and body fat as measured through imaging.

The prospective nature of UK Biobank as well as the large sample size has enabled a large number of incident events to be captured through cohort-wide follow-up, including ∼40 000 incident cancers, ∼14 000 deaths and 1.3 million hospitalizations, a substantial number of which are attributable to cardiovascular disease. By end of March 2015, there were 5800 incident cases of myocardial infarction and 3600 incident cases of stroke using an adjudicated algorithm that incorporates self-report, death and hospital inpatient data. When primary care data are made available for the full cohort, it is anticipated that the number of cases will increase by 10–15% for myocardial infarction and 50% for stroke. This linkage will not only aid the ascertainment of certain conditions underdiagnosed in a hospital inpatient setting, but will also provide information on laboratory and physical measurements, referrals, and prescriptions. The large number of events allows the exploration of well-powered exposure-outcome associations as well as the development and/or validation of risk prediction models.^26^

## Accessing UK Biobank data

UK Biobank is an open-access resource which any bona fide researcher can apply to use (without the need for collaboration with UK Biobank scientists), to conduct health-related research that is in the public good. Applications to use the UK Biobank resource can be made from researchers from the academic, commercial, charity, or public sector, from either the UK or internationally, with access being granted on a non-preferential and non-exclusive basis. Once researchers have registered with UK Biobank (www.ukbiobank.ac.uk), they can submit an application, which involves a brief description of the scientific rationale, aims, methodology and expected value of the project. Researchers select the required data-fields through the data showcase (www.ukbiobank.ac.uk/data-showcase/), which provides complete information on each variable available, how it was collected and the univariate distribution of participants across categories. Following approval by the Access Sub-Committee, researchers are required to sign a Material Transfer Agreement before downloading the data.

Applications can be for data only, sample requests or proposals to re-contact participants. The main requirement for data only applications is that the scope of the application can be clearly defined. Applications can be hypothesis-driven or hypothesis generating, involve a range of phenotypic/genotypic data or be focused on developing novel methods. Applications that request biological samples are subject to higher levels of scrutiny due to the depletable nature of the resource; researchers therefore need to provide a strong scientific justification together with assay details and sample requirements. UK Biobank also welcomes proposals to recontact participants for participation in other research studies, although these are also carefully scrutinized and their implementation needs careful management to avoid over-burdening participants.

Researchers are required to publish their results in an academic journal or an open source publication site (e.g. bioRxiv) and to return their findings (i.e. the underlying code used to generate the findings and any derived variables that were generated as part of the research) to UK Biobank so that these can be made available to share with other researchers.

## Research interest and output

Between April 2012, when UK Biobank was opened for research use, and April 2017, 4600 researchers had registered to use the resource, >880 applications had been submitted and 430 projects were ongoing. Since 2013, there has been a three-fold increase in applications, with a particular increase from international researchers (9% in 2013, 23% in 2014, 44% in 2015 and 59% in 2016), predominantly from the USA (16% of total applications) and mainland Europe (14% of total applications), reflecting increasing global awareness of UK Biobank as a major resource for health-related research.

All approved research projects, including a short description of their objectives, can be found in the following searchable database: http://www.ukbiobank.ac.uk/approved-research/. More than 100 applications are focused on ‘cardiovascular disease’, which is, to date, the most common health outcome of research interest in UK Biobank. The vast majority of applications have been ‘data only’ requests (>95%), although projects are now underway that have requested biological samples (e.g. for exome sequencing or copy number variant measurement) and, to a lesser extent, to re-contacting participants to invite them to join other research studies.

The resource is still in its relatively early stages as regards research output, but the number of publications and conference abstracts based upon UK Biobank data are steadily increasing (http://www.ukbiobank.ac.uk/published-papers/). By January 2017, more than 130 peer-reviewed journal articles had been published, including several within the area of cardiovascular research. These have mainly involved cross-sectional associations between traditional risk factors and cardiovascular disease.^27–29^ However, as more incident cases accrue, research will begin to take advantage of the prospective nature of the cohort.

Genotyping data for the first 150 000 participants became available in November 2015, and results using this data are beginning to emerge. For example, a recently published study found that a subset of ‘favourable adiposity’ alleles associated with higher likelihood of adiposity were in turn associated with a lower risk of hypertension and heart disease.^30^ Several genome-wide association studies have also identified novel genetic variants linked with blood pressure phenotypes.^31^^,^^32^ The genotyping data for all 500 000 participants will be released during 2017, and a corresponding increased interest from the research community in using the genetic data is expected.

## Conclusion

Observational cohort studies have been essential in informing the prevention and treatment of cardiovascular diseases and identifying the role of cardiovascular risk factors in disease development. However, previous cohorts have either been too small to investigate less common diseases or lacked the depth of data to explore the complex interplay between different factors and cardiovascular disease risk. UK Biobank combines a large sample size of half a million participants with an unprecedented amount of phenotypic and genotypic data as well as ongoing linkage to health records. This open-access resource provides researchers worldwide with the opportunity to address a wide variety of novel research questions with the aim of improving the prevention, treatment and diagnosis of cardiovascular disease.

## Acknowledgements

Special thanks to Associate Professor Sarah Lewington for providing *Figure **1* which contains data from the Prospective Studies Collaboration and to Paul Sherliker for generating the figure. Acknowledgements to the members of the UK Biobank Steering Committee; Prof. John Danesh, Prof. Paul Elliot, Prof. John Gallacher, Prof. Jane Green, Prof. Paul Matthews, Dr Tim Peakman and Prof. Jill Pell. Additional thanks to the UK Biobank Access team (Lorraine Gillions, Erin Scobie, Tobie Rhyman, Rick Hayward, Paul Flood, Louise Taylor and Danielle Duff) for their tireless work on research registrations, applications and output and providing this information for the article.

## Funding

UK Biobank was established by the Wellcome Trust; Medical Research Council; Department of Health and the Scottish Government. UK Biobank has also received funding from the Welsh Assembly Government; British Heart Foundation and Diabetes UK.

**Conflict of interest:** none declared.
